# Fluoroalcohols for chemical modification of biomolecules

**DOI:** 10.1016/j.tchem.2024.100088

**Published:** 2024-08-06

**Authors:** Mohammad Nuruzzaman, Zeinab M. Nizam, Jun Ohata

**Affiliations:** Department of Chemistry, North Carolina State University, Raleigh, NC, 27695, United States

**Keywords:** Bioconjugation, Trifluoroethanol (TFE), Hexafluoroisopropanol (HFIP), Polypeptide, Nucleic acid, Saccharide

## Abstract

While their broad utility in various chemistry fields were well recognized for decades, fluoroalcohols have recently emerged as a unique solvent system for bioconjugation development. This review describes examples and roles of fluoroalcohols such as trifluoroethanol (TFE) and hexafluoroisopropanol (HFIP) for chemical modification of biomolecules such as polypeptides, nucleic acids, and saccharides. Many chemical modification processes were facilitated by notable functions of those fluoroalcohols such as a proton shuttle, reversible adduct formation with reactive species, and compatibility with electrochemistry/photochemistry. The usefulness of the fluoroalcohol solvents can be even promoted by its combination with a different solvent system for reaction enhancement and protein stabilization. The collection of the various chemical transformations in this review is an indication of the rapid growth of the solvent-assisted bioconjugation field.

## 1. Introduction

Modern chemical bioconjugation technologies take advantage of a variety of chemical strategies such as use of unique solvents to address the reactivity and selectivity challenges of the process. Chemical modification of biomolecules or bioconjugation is one of the applications of organic chemistry, which require selective chemical transformation to occur in a controlled manner. For example, inspired by sophisticated chemical systems of nature, enzyme-mediated methods offer high reaction efficiency and selectivity for bioconjugation purposes [1]. Unique molecular design can be also utilized for effective nonenzymatic bioconjugation including chemoselective labeling agents [2], photocatalysis [3], and proximity-driven chemistry [4]. Although aqueous media are often considered ideal for bioconjugation processes, nonaqueous systems can be of great use for certain biomolecule functionalization purposes [5,6], and this review is focused on recent development of bioconjugation techniques using trifluoroethanol (TFE) and hexafluoroisopropanol (HFIP) as a reaction medium.

**Fig. 1.**
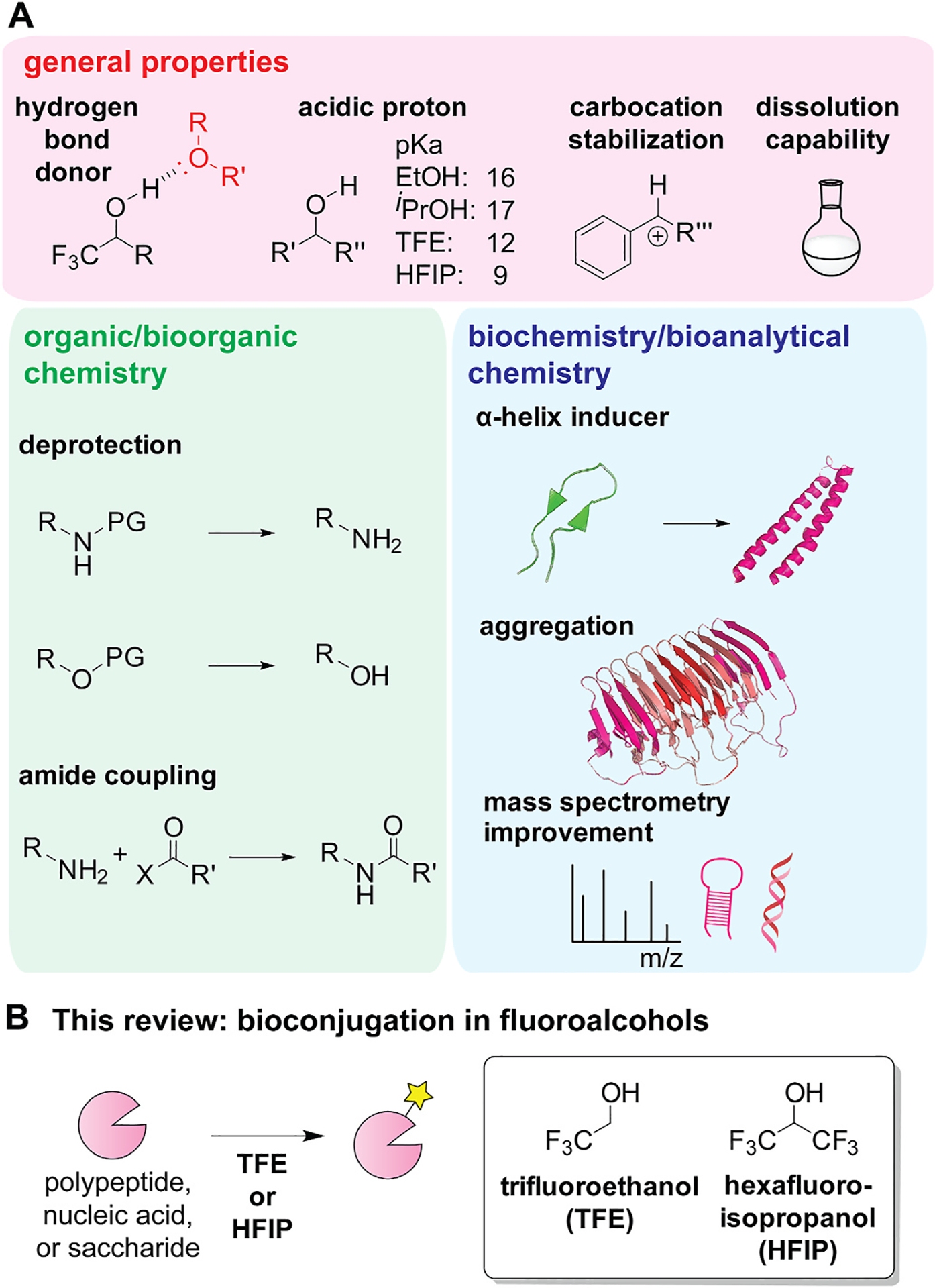
Fluoroalcohols such as trifluoroethanol (TFE) and hexafluoroisopropanol (HFIP) in chemistry and biology fields. (A) Depiction of general properties of TFE and HFIP and examples of traditional use of TFE and HFIP for organic/bioorganic synthesis as well as biochemistry and bioanalytical chemistry fields. PG: Protecting group. (B) Theme of the review: bioconjugation of polypeptides, nucleic acids, and saccharides in TFE and HFIP.

Because of their unique attributes, synthetic organic chemistry as well as bioorganic chemistry have been using TFE and HFIP as media for deprotection and condensation reactions [7]. The fluoroalcohol solvents are known to exhibit distinct functions from typical alcoholic solvents. The fluorine atoms make the hydroxyl group serve as an excellent hydrogen bonding donor and become more acidic (Fig. 1A). The presence of fluorine atoms enables stabilization of carbocation species and exceptional dissolution capability. One of the traditional uses of such fluoroalcohols is for mild deprotection of acid-sensitive functional groups in small molecules by leveraging their acidic nature (Fig. 1A). Another common use of the fluoroalcohols is for amide coupling reactions, and use of fluoroalcohol solvents for small molecule synthesis are extensively reviewed in literature [8–10].

In addition to the reaction enhancement, TFE and HFIP demonstrated their utility in biochemical and bioanalytical fields (Fig. 1A) [11, 12]. Those fluoroalcohols are known to be α-helix inducer that causes alteration of secondary structure from a native one in an aqueous solution. At the same time, aggregation of polypeptides can be exerted by the solvent systems, and the aggregation-inducing ability has been used for study of certain peptides such as amyloid-β [13]. Finally, mass spectrometry analysis of oligonucleotide samples is commonly done in the presence of HFIP conducive for ionization of the poly anionic biomolecules [14,15].

This review article describes recent advances of chemical modification strategies of biomolecules using TFE and HFIP (Fig. 1B). There has been an increasing number of bioconjugation methods in those fluoroalcohol solvents recently, and we particularly highlighted publications during the past five years. The organization of the review is based on biomolecule types (i.e., polypeptides, nucleic acids, and saccharides). The bioconjugation schemes in the sections are accompanied by images underscoring the importance of the fluoroalcohols in each system (e.g., comparison with other solvents and roles of solvents in a transition state).

## 2. Polypeptide modification

While there are many examples of traditional use of fluoroalcohols for polypeptide chemistry (e.g., deprotection/cleavage reactions [16, 17], amide coupling reactions [18,19], and substitution reactions [20]), the following sections of polypeptide bioconjugation in fluoroalcohols are focused on reports utilizing distinct chemical transformations and organized based on types of amino acid involved in a given labeling process. It is obvious that the recent examples are particularly focused on targeting aromatic amino acid residues (Tyr and Trp) [21] as well as lysine residues by harnessing the capabilities of the fluoroalcohols.

### 2.1. Tyrosine and tryptophan modification

**Fig. 2.**
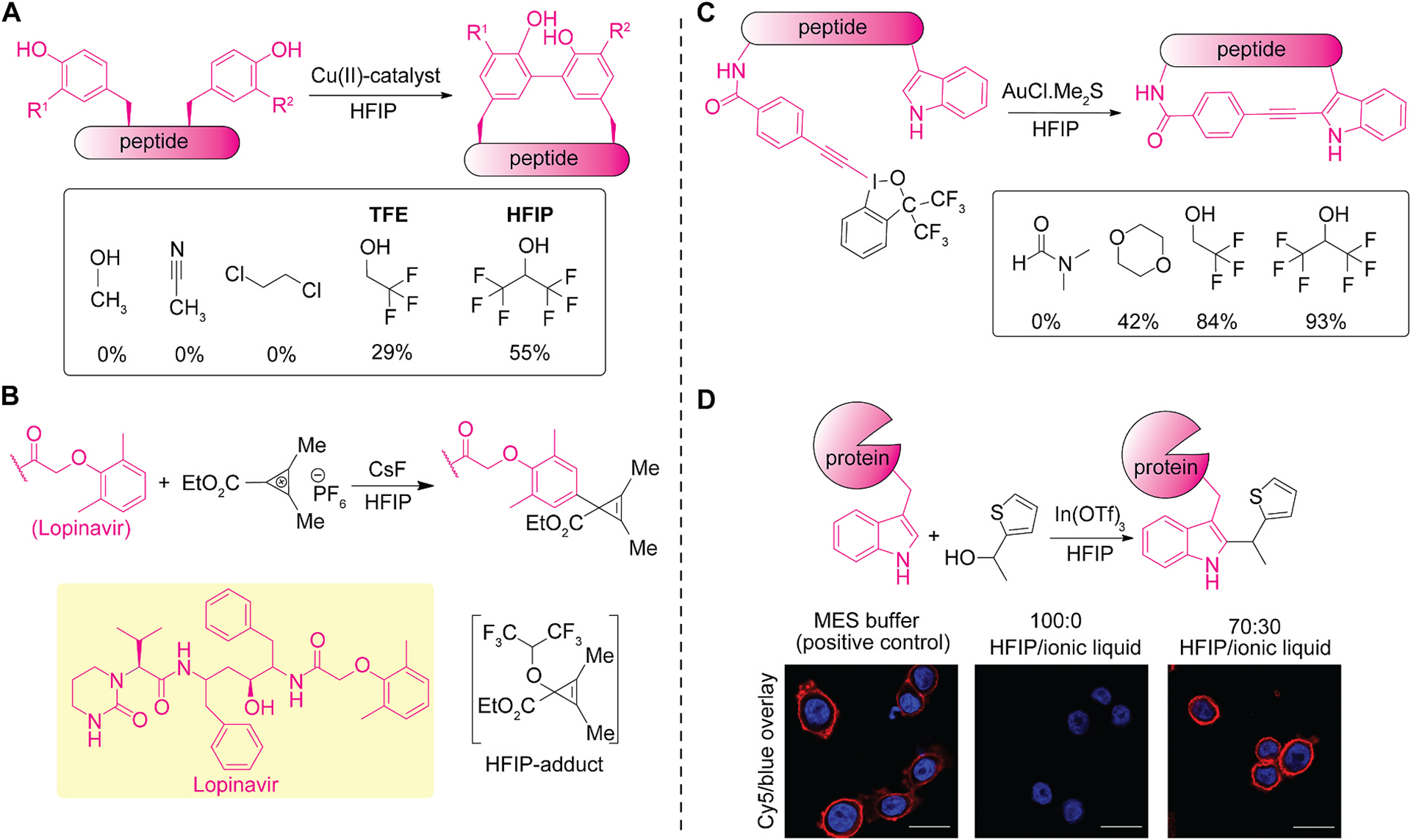
Tyrosine and tryptophan modification in fluoroalcohols such as hexafluoroisopropanol (HFIP). (A) Oxidative coupling of tyrosine residues using copper(II) catalyst. The numbers below the solvents in the box below the scheme showing high-performance liquid chromatography (HPLC) conversion of modification reactions. (B) C–H cyclopropenylation of a synthetic peptide Lopinavir bearing an alkoxyaryl unit. (C) Gold(I)-catalyzed C–H alkynylation of tryptophan residues of peptides, which generates a fluorescent peptide useable for live-cell imaging. The numbers below the solvents in the box showing HPLC conversion of modification reactions. (D) Dehydrative C–H alkylation of tryptophan residues of peptides and proteins in fluoroalcohols. Confocal microscopy images of breast cancer cells (SK-BR-3) stained with Herceptin antibody and visualized by anti-human secondary antibody — Cy5 conjugate (red) and nuclear stain (DAPI, blue). Scale bars: 20 μm. Herceptin was pretreated in (*N*-morpholino)ethanesulfonic acid (MES) buffer or a mixture of ionic liquid (1-ethyl-3-methylimidazolium tetrafluoroborate)/HFIP. The images of confocal microscopy was reproduced with permission [26]. Copyright 2024, American Chemical Society.

An oxidative macrocyclization between two tyrosine residues can be catalytically induced by a multicopper(II) cluster using fluoroalcohol solvents (Fig. 2A) [22]. Oxidative coupling of two tyrosine residues are known [23], and this report utilized multicopper(II) cluster-catalyzed intramolecular macrocyclization on tyrosine residues to form a biaryl linkage. Hexafluoroisopropanol (HFIP) plays a crucial role in the formation of copper clusters necessary for the catalytic transformation. Additionally, solvent screening revealed HFIP as the optimal solvent for the reaction, surpassing other fluoroalcohols such as trifluoroethanol/TFE (29%), 1,1,1-trifluoropropan-2-ol (8%), and perfluoro-*tert*-butanol (2%). When the reactions were carried out in typical organic solvents (e.g., methanol, acetonitrile, and 1,2-dichloroethane), only negligible product formation was observed. The report demonstrated that this macrocyclization process tolerates some functional groups on the peptide including alkyl (e.g., Ala and Val) and ester, which is perhaps indicative of incompatibility with other canonical amino acids.

Fluoroalcohols promote the cyclopropenylation of electron-rich aromatic systems through stabilization/interaction with the active cationic reagent (Fig. 2B) [24]. The reaction proceeds through the Friedel-Crafts type alkylation, where the cyclopropenium reagent exhibits a preference for reacting with aryl C–H bonds over other functional groups such as carboxylic acid, ester, ketone, amide, alcohol, alkene, and secondary amines in substrates. The model reaction of the reagent with *p*-xylene showed the superior nature of HFIP to other solvents including TFE and dimethylformamide (DMF). This observation is consistent with many studies utilizing HFIP for Friedel-Crafts alkylation, where the solvent’s cation stabilization effects play a crucial role [7]. Furthermore, an HFIP-bound cyclopropene was detected and could be an important species during the reaction, showcasing the potential role of HFIP in reversibly masking the reactive intermediate for minimizing side reactions. Further organic transformations can utilize the incorporated cyclopropene moiety on the substrate to access a variety of cyclic moieties, such as furan and naphthol. Additionally, this method successfully achieved the cyclopropenylation of other types of biomolecules like cholesterol.

Gold-mediated intramolecular arene-alkyne coupling can be enhanced in HFIP as a reaction solvent for the formation of a macro-cyclic fluorescent peptide (Fig. 2C) [25]. This process involves intramolecular C–H alkynylation between a tryptophan residue and an unnatural amino acid bearing a hypervalent iodine moiety using a gold (I) catalyst. The hypervalent iodine moiety is installed by selective amidation on lysine or the N-terminus, which then reacts with tryptophan residues on the peptide chain forming the N-terminal–tryptophan or lysine–tryptophan cyclization product. Fluoroalcohols, particularly HFIP, demonstrated improved performance in comparison to other solvents including methanol, acidic acetonitrile, and dioxane. Additionally, the benziodoxole reagent bearing two trifluoromethyl group (i.e., hexafluoroisopropyl motif) exhibited a higher cyclization efficiency than that without the fluoroalkyl groups, implying the significance of the fluoroalkyl scaffolds even in the reagent design. Though the indole-alkyne bond forming process is compatible with most of the twenty canonical amino acids, *tert*-butyl version of thioether on methionine was necessary to achieve high conversion possibly due to methyl thioether coordination to the metal catalyst. The resulting alkyne-tryptophan linked cyclic peptide possesses fluorescent properties, enabling live-cell imaging without the need for additional fluorophore groups.

Tryptophan-selective, dehydrative alkylation of peptides and proteins can be achieved in HFIP and potentially compatible with the biomolecules with the aid of ionic liquids (Fig. 2D) [26]. This process requires thiophene-ethanol labeling reagent and a Lewis acid catalyst (e.g., indium triflate) that undergoes bond formation with the indole group of tryptophan residues through dehydration process. Mechanistic investigations using density functional theory (DFT) calculations indicated that the synergistic effect of the Lewis acid and HFIP facilitates thiophene-ethanol dehydration, generating a benzylic cation intermediate, as hydrogen bonding and cation stabilization ability of HFIP have also been reported in similar Friedel-Crafts alkylation-type reactions [27–29]. A series of proteins, including a monoclonal antibody, has been functionalized using the alkylation method, where that the labeling agent chemoselectively modified the indole group of tryptophan over other amino acid residues in the peptide chain containing virtually any of the canonical amino acids. Conservation of antibody activity after treatment in a mixture of HFIP and ionic liquid was also demonstrated through Western blot and an immunofluorescence assay of breast cancer cell lines. While this work showed examples of compatibility of HFIP with only two proteins (lysozyme and Herceptin antibody), it may be possible that modification of proteins can be generally performed in fluoroalcohol solvents through stabilization of protein structure by ionic liquid additives. Applications of bioconjugation methods to protein substrates are of value in numerous purposes, and development of such stabilization strategies of proteins in a fluoroalcohol mixture may be key for the further growth of the field.

### 2.2. Lysine modification

**Fig. 3.**
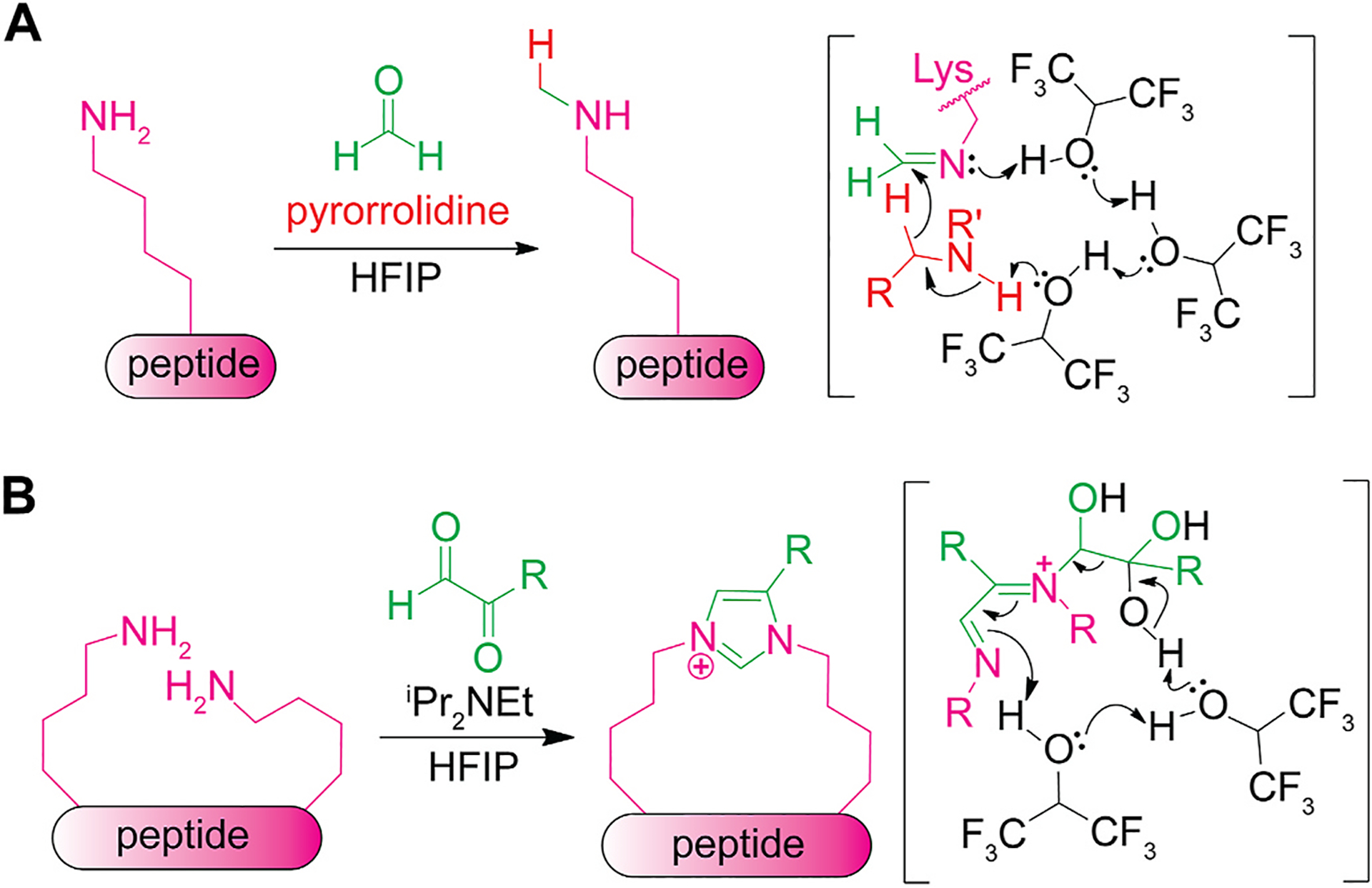
*N*-methylation and macrocyclization of lysine (Lys) in hexafluoroisopropanol (HFIP). (A) Use of HFIP as a proton shuttle during the *N*-methylation of lysine residues of peptides with formaldehyde. (B) Cyclization of lysine residues of peptide with α-ketoaldehydes in HFIP.

Formaldehyde-mediated *N*-methylation of lysine residues in peptides can be facilitated by HFIP through a putative proton shuttle (Fig. 3A) [30]. Lysine methylation naturally occurs as epigenetic regulation of cellular functions including its influence on transcription through histone methylation [31,32]. This report by Chen and co-workers was a chemical mono-methylation method of lysine residues in peptide using formaldehyde in HFIP. The mechanism involves hydride transfer from a secondary amine additive to an imine intermediate formed from lysine and formaldehyde. The energetically constrained hydride transfer was proposed to be made feasible through a proton shuttle transition state where a cluster of HFIP assist transfer of hydrogen atoms. Fluoroalcohols like HFIP and TFE were found to be effective whereas typical organic solvents displayed minimal or no formation of the alkylated product. The selective *N*-methylation process is compatible with most of the side chains of canonical amino acids except for tryptophan (indole), arginine (guanidine), and cysteine (thiol). Modulation of reaction conditions can alter the reaction product of the method from the methylated amines to α,β-unsaturated carbonyls through oxidative deamination processes, which can be further modified with nucleophiles such as hydrazone, thiol, and amines.

HFIP enabled the crosslinking of two lysine residues of a peptide and α-ketoaldehyde reagent, which was also proposed to be accelerated through a proton shuttle mechanism (Fig. 3B) [33]. The reaction involves the formation of an imidazolium linker by coupling two lysine residues with α-ketoaldehyde. Though prior literature reported linkage formation between lysine and arginine in aqueous media in a similar reaction condition [34], this system with HFIP did not induce reactions on the guanidine group of arginine residues. Mechanistically, the imidazolium formation was proposed to initiate with the generation of two imine/iminium groups first, and then HFIP-mediated consecutive hydrogen transfer was proposed to lead to the product formation. Fluoroalcohols, particularly HFIP, were identified as the optimal solvent. In contrast, solvents such as methanol, acetonitrile, DMF, and aqueous solutions showed minimal conversion. This amine-selective coupling reaction tolerates the functional groups in all the proteogenic amino acid residues except cysteine (thiol groups).

### 2.3. Other amino acid modification

**Fig. 4.**
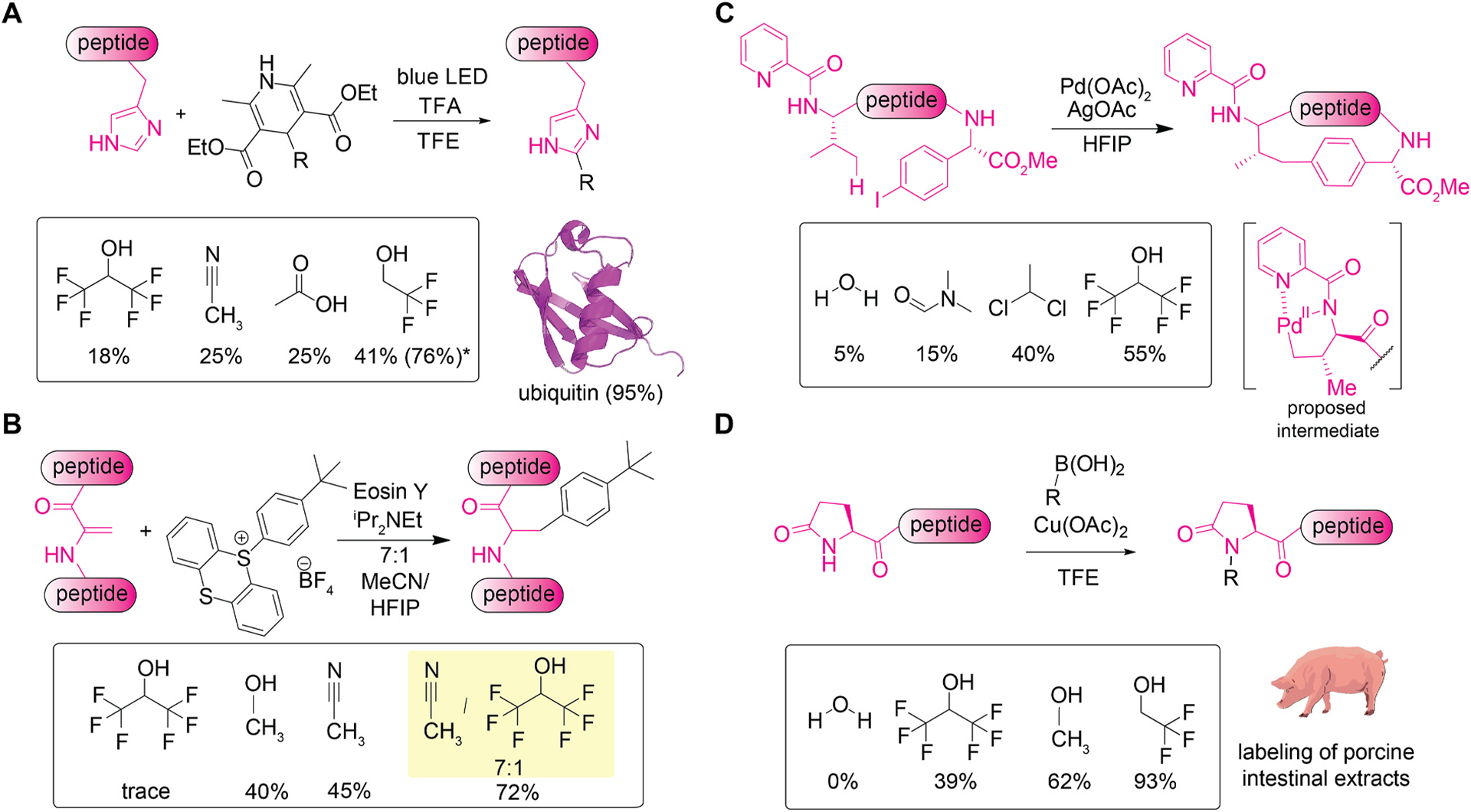
Modification of histidine, dehydroalanine, valine, and pyroglutamate in fluoroalcohols such as trifluoroethanol (TFE) and hexafluoroisopropanol (HFIP). (A) Photochemical alkylation of histidine residues in peptides. The numbers below the structures in a box showing liquid chromatography (LC) conversion of peptide modification for the solvent screening. *Conversion in the parenthesis is after three cycles of the alkylation reaction. PDB ID for the ubiquitin structure: 1UBQ. (B) C–H arylation of dehydroalanine containing peptides using a sulfonium salts in HFIP. The numbers below the structures in a box showing isolated yields of products. (C) Pd-catalyzed intramolecular C–H arylation of N-terminal valine residue of peptide with aryl iodides. The numbers below the structures in a box showing LC conversion of peptide modification for the solvent screening. (D) Copper(II)-catalyzed arylation and alkenylation of pyroglutamate with boronic acid reagents. The numbers below the structures in a box showing LC conversion of peptide modification for the solvent screening.

Light-mediated alkylation reactions of histidine residues proceed in a particularly efficient fashion by using TFE solvent (Fig. 4A) [35]. Development of histidine-targeting labeling methods was a challenging task [36–38], and this radical reaction takes advantage of a photo-reactivity of a labeling reagent, dihydropyridine derivative, to achieve the chemoselective bioconjugation. One of the key steps in the proposed reaction mechanism was the addition of alkylradical to protonated imidazole generating a radical cation intermediate, and the potential importance of fluoroalcohol solvents for the protonation and cation stabilization processes can be inferred. Indeed, a reaction of a model substrate in TFE offered the highest conversion, compared to other acidic solvents such as HFIP and acetic acid. The method was amenable to a variety of unprotected peptide substrates including ubiquitin containing 76 amino acids.

The photocatalytic arylation of dehydroalanine residues in peptides was enabled by a mixture of HFIP and acetonitrile solvents with use of a sulfonium reagent and organic photocatalyst (Fig. 4B) [39]. Dehydroalanine is a product of post-translational modifications and is usually formed from a serine and cysteine residue through elimination processes [40]. The light-induced arylation was likely to be driven by a single-electron transfer activating the sulfonium reagent to generate reactive aryl radicals that reacts with the α,β-unsaturated carbonyl group, as such reactivity is often observed for dehydroalanine [41]. Modest and moderate conversions into the arylated product were observed for many solvents such as HFIP (trace), DMF (trace), dichloromethane (15%), methanol (40%) and acetonitrile (45%). On the other hand, use of a binary mixture of acetonitrile and HFIP (7:1 ratio) gave a conversion of 72 %. Although the arylation reaction is compatible with several functionalities such as thioether, phenol, and indole groups on substrates, tested polypeptides were relatively small (≤6-mer peptide).

Ligand-directed Pd-catalyzed intramolecular arylation with aryl iodide is possible at the terminal alkyl carbon of a valine residue in HFIP (Fig. 4C) [42]. By incorporating iodophenylalanine and a pyridine-based group on an N-terminal valine residue, arylation occurred selectively at the methyl group of the valine residue forming a peptide macrocycle. The proposed reaction mechanism begins with the coordination of palladium (0) to a pyridyl-amide group, followed by C–H palladation with the γ-methyl group of the valine residue, oxidative addition of iodophenylalanine, and reductive elimination to form the arylated product. Potentially due to the dissolution ability, fluoroalcohols like HFIP offered relatively high conversion (55–76 %), and in contrast, moderate or modest conversions were observed for 1,1-dichloroethane, DMF, and water. The reaction is capable of formation of a large macrocycle (36-membered ring) but its incompatibility with certain amino acid residues such as cysteine and histidine was indicated in the report.

TFE was shown to be capable of enhancing chemical modification of pyroglutamic acid through copper(II)-catalyzed arylation/alkenylation chemistry (Fig. 4D) [43]. Pyroglutamic acid or pyroglutamate is an N-terminal, cyclic amino acid that would be generated through post-translational modification process and is often found in peptide hormones [44]. Pyroglutamate-histidine sequence was previously shown to be reactive toward Chan-Lam coupling processes through binding of a copper ion to the peptide backbone and histidine imidazole [45]. While the preceding work of the histidine-directed chemistry was more efficient in aqueous media, this work in TFE proceed without the aid of histidine, serving as a general pyroglutamate detection method. The reaction can proceed by using HFIP and methanol as solvents in a less efficient way than TFE. In stark contrast, aqueous buffer and other alcohols such as ethanol and isopropanol would not provide any observable amount of the product, underscoring the importance of the fluoroalcohol solvent and difference from the previous histidine-directed chemistry. Solvent roles for the reaction have not been elucidated, but perhaps the necessity of the fluoroalcohol may be related to their interaction/reactivity to the boronate reagent and copper catalyst. The method has been applied to labeling of porcine intestinal extract to label pyroglutamate-containing peptides in a complex mixture.

## 3. Nucleic acid and saccharide modification

Compared to the polypeptide bioconjugation in the previous section, there have been substantially fewer reports of chemical modification of nucleic acids and saccharides in fluoroalcohols. To our knowledge, fluoroalcohols have been unexplored for bioconjugation of oligonucleoside/oligonucleosides—except the traditional deprotection process for the DNA-encoded library [46,47]— as well as for oligosaccharides. The examples of in the following sections are chemical modification of small biomolecule building blocks (distinct from the deprotection reactions [48,49]), and those reactions have tackled the reactivity and selectivity challenges of the labeling processes.

### 3.1. Nucleic acid modification

**Fig. 5.**
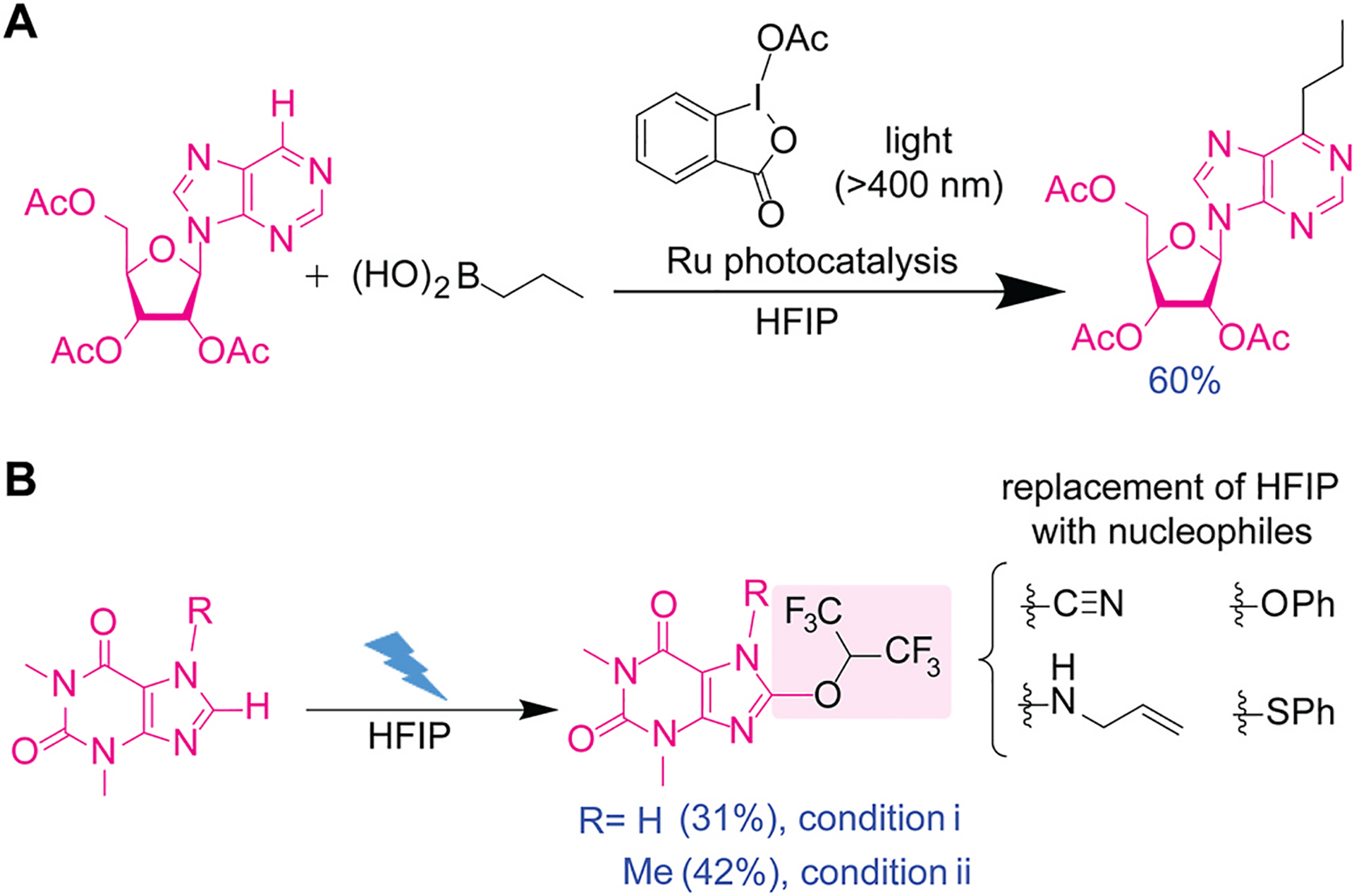
Functionalization of purine-based substrates in hexafluoroisopropanol (HFIP). (A) Photoredox Minisci C–H alkylation of purine riboside substrate with propylboronic acid in HFIP. Ru photocatalysis: tris(bipyridine)ruthenium(II) chloride with >400 nm light. (B) Electrochemical C–H functionalization of caffeine (R=Me) and theophylline (R=H) using HFIP. Condition (i): 7.2 mA/cm^2^, 2.0 F, 5 h. Condition (ii): 22.1 mA/cm^2^, 2.61 F, 1.75 h.

Ruthenium-catalyzed photoredox alkylation of nucleoside derivatives can be successfully achieved in hexafluoroisopropanol (HFIP) using alkylboronic acid and hypervalent iodine reagents (Fig. 5A) [50]. Upon irradiation of visible light (>400 nm), ruthenium catalyst [Ru (bpy)_3_]Cl_2_ or tris(bipyridine)ruthenium(II) chloride would induce single-electron transfer (SET) that reduces the hypervalent iodine reagent (acetoxybenziodoxole), generating an iodine-based radical intermediate. The iodine-centered radical was proposed to produce an alkyl radical derived from the boronic acid reagent, which eventually would be added to the *N*-heteroarene substrates. For instance, a purine riboside substrate was alkylated with propylboronic acid. Solvent screening using a model substrate, 4-chloroquinolone, showed that HFIP was crucial for obtaining a good yield (88 %), compared to other solvents like dichloromethane (33 %) and acetonitrile (38 %). The reaction could proceed even without the photocatalyst by relying on another radical-based mechanism, which may be potentially relevant to known HFIP-mediated reactions of H_2_O_2_ [7]. Although the DFT calculation of reaction mechanism did not indicate potential involvement of HFIP, it is known that HFIP enhances the reactivity of boronic acid for various chemical transformations such as Beckmann rearrangement by increasing the electrophilicity and Lewis acidity of the boron center [51, 52]. Furthermore, HFIP can enhance hypervalent iodine chemistry during the nucleophilic substitution, oxidative cyclizations, and aromatic C–H amination processes [53–55], and collectively, HFIP could be playing multiple roles in this labeling reaction.

Electrochemical formation of aryl-hexafluoroisopropyl ether on purine derivatives proved feasible in HFIP, which is useful for secondary functionalization with other nucleophiles (Fig. 5B) [56]. Ether formation on the purine scaffold of caffeine and theophylline compounds was enabled by a boron-doped diamond electrode for the electrochemical reaction, as the electrode was also effective on a different electrochemical reaction of arenes [57]. Moreover, the HFIP moiety on the heteroarene ring after the labeling process serves as a leaving group, facilitating further functionalization of the ether through nucleophilic aromatic substitution processes. The secondary modification allows for the introduction of various functional groups, including amine- and thiol-derived motifs, demonstrating the utility of the arene-hexafluoroisopropyl ether formation reaction. This method seems to require methyl groups to protect some of the NH groups of the heteroarene system, and further improvement may be necessary to utilize the method for modification of unprotected nucleoside/nucleotide derivatives.

### 3.2. Saccharide modification

**Fig. 6.**
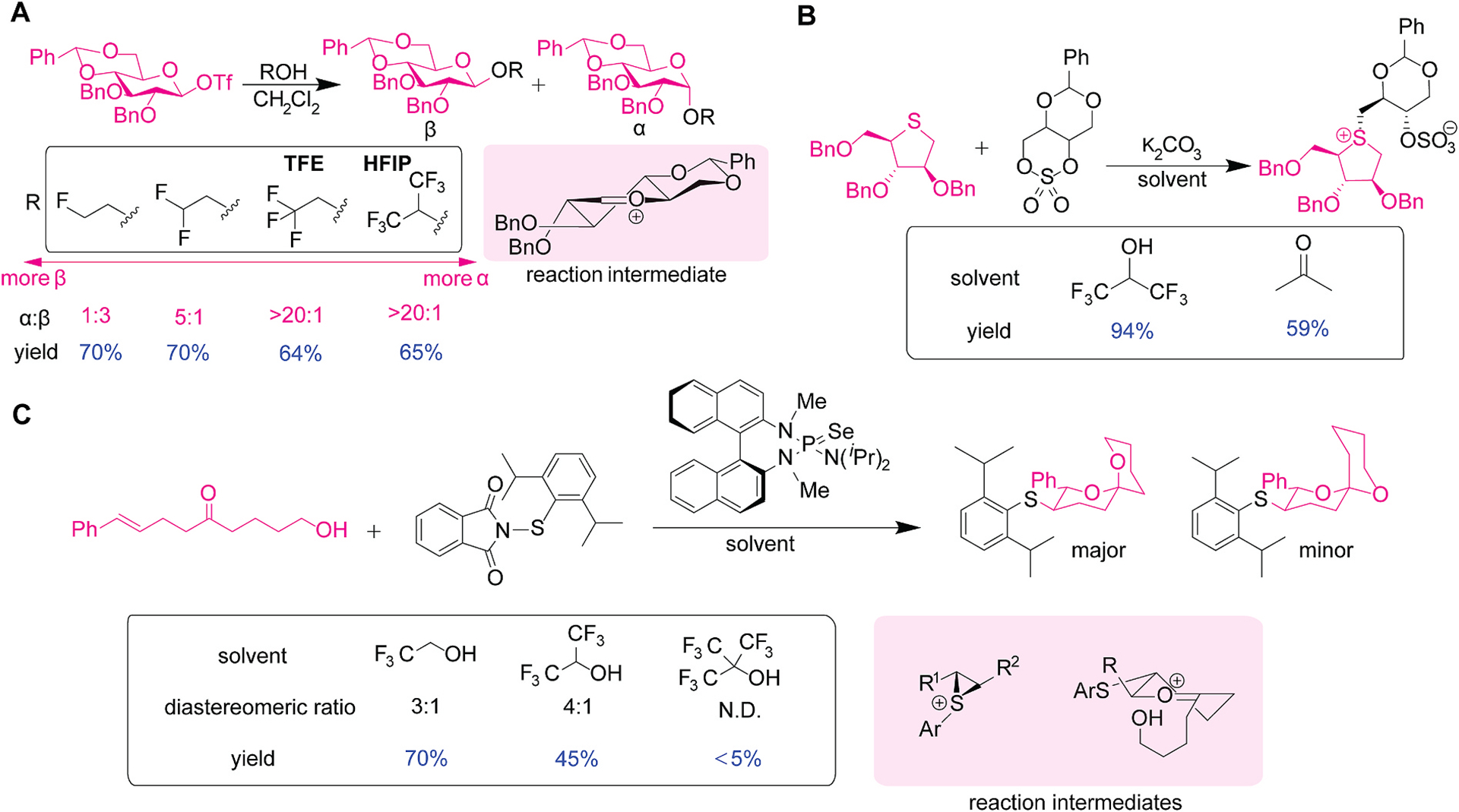
Modification of saccharide substrates in trifluoroethanol (TFE) and hexafluoroisopropanol (HFIP). (A) *O*-alkylation of glucose derivatives with a set of alcohol nucleophiles. The structure of the reaction intermediate is shown at the right bottom. Box showing the diastereomeric ratio and yield determined by NMR spectroscopy. (B) *S*-alkylation reaction of sulfur-based saccharides with sulfate reagents in HFIP and acetone. Box showing the yield of isolated product. (C) Lewis base-catalyzed spiroketalization reaction of unsaturated hydroxy ketone substrate using a set of fluoroalcohol solvents and the reaction intermediates during spiroketal formation mechanism. Box showing the ratio of major and minor diastereomers and isolated yield. N.D.: Not determined.

Trifluoroethanol (TFE) and HFIP could act as a weak nucleophile and offer unique selectivity for *O*-alkylation of saccharide derivatives (Fig. 6A) [58]. The study by Codée and co-workers demonstrated stereochemical controls of *O*-alkylation of a protected saccharide based on nucleophilicity of alcohol reagents. For instance, whereas alkylalcohols (e.g., ethanol) and monofluorinated alkylalcohols favored the β addition product, HFIP and TFE showed substantial preference to the α product. The authors proposed mechanistic pathways to explain the observed stereochemical outcomes. Strong nucleophilic alcohols tend to undergo a concerted replacement mechanism (S_N_2-type), while weak nucleophiles like TFE and HFIP facilitate cationic intermediate formation through an S_N_1-type mechanism. The study also highlights the potential importance of fluorinated alcohols in interacting with or stabilizing cationic intermediates, as acylium ions are proposed as a potential reaction intermediate.

HFIP is a useful solvent for *S*-alkylation reaction of sulfur-based saccharide derivatives to access a class of sulfonium-ion glucosidase inhibitors (Fig. 6B) [59]. Salacinol is a naturally occurring sulfur-containing saccharide with a sulfur atom incorporated into the ring and acts as a potent α-glucosidase inhibitor used for diabetes treatment [60]. The relatively higher nucleophilicity of thioether-containing saccharides would be expected compared to typical oxygen-based saccharides, which is somewhat similar to the case of methionine in proteins [61]. The use of HFIP as a solvent for the reaction of sulfur-based saccharide resulted in higher yields of alkylated product (94 %) that that of acetone (59 %), likely due to the enhanced solvation of transition states and potential adduct formation in the HFIP system. This observation may be hinting at the potential utility of fluoroalcohols for other thioether bioconjugation chemistry applications.

Lewis base-catalyzed cyclization reactions in fluoroalcohol solvents was developed, leading to the formation of spiroketals that are akin to saccharide structures (Fig. 6C) [62]. The reaction utilized a chiral Lewis base, selenophosphoramide and phthalimide-based reagent. Fluoroalcohol solvents including TFE, HFIP, and perfluorinated-*tert*-butanol were screened for the spiroketalization reaction, showing that HFIP was found to offer better diastereoselectivity (4:1) and modest yield (45 %) compared to other solvents. In contrast, TFE provided the highest yield (70 %) but with a lower diastereoselectivity of 3:1. Nonafluoro-*tert*-butanol was not an effective solvent for this reaction producing a trace amount of the product, which was ascribed to the poor solubility of the reagents in this solvent system. TFE and HFIP may play a role in potential stabilization of various cationic intermediates throughout the reaction processes (e.g., stabilization of sulfenium and oxocarbenium ions).

## 4. Conclusion

A number of recent reports demonstrated that trifluoroethanol (TFE) and hexafluoroisopropanol (HFIP) are capable of various modification reactions of biomolecules. As highlighted in this review, the emergence of these important reports within the past few years may be indicative of the power of those fluoroalcohol solvents enabling profound chemical transformation. Yet, the fluoroalcohol-enhanced biomolecular reactions probably are in its nascent state, as technologies for many biomolecules or biomacromolecules in those fluoroalcohols are still virtually nonexistent including fatty acid/lipid labeling (though labeling of cholesterol derivatives was achieved by a method described in Fig. 2B [24]), oligonucleotides, and oligosaccharides. Examples of photochemical and electrochemical approaches (e.g., Fig. 5) may indicate that those strategies could be expanded to labeling of other biomolecules as well. Indeed, electrochemistry has been demonstrated to be conducive for redox-innocent hexafluoroisopropyl anion generation recently [63], and actions of unique chemical species in fluoroalcohols may be key for future development of the area. Enzymatic bioconjugation in fluoroalcohols has not been reported presumably due to the known denaturing effects of those solvents, but ionic liquid stabilization of proteins in HFIP may be of help in this context [26]. Computational studies of fluoroalcohols represents formidable challenges [64], especially with a large biomolecule system, and rational design of new bioconjugation reactions in the future may depend on further improvement on the computational strategies. Overall, chemical diversity of bioconjugation reactions in fluoroalcohols has begun to expand promptly and is likely to remain critical in various field of chemistry and biology in the next decades.

## Data Availability

No data was used for the research described in the article.
